# Aptamers as Potential Inhibitors of Ethylene Biosynthesis: Identification and In Silico Selection

**DOI:** 10.3390/ijms26178146

**Published:** 2025-08-22

**Authors:** Diana Laura Aparicio-Breceda, Cristian Patricia Cabrales-Arellano, Efren Delgado, Gerardo Antonio Pámanes-Carrasco, Jorge Iñaki Gamero-Barraza, Hiram Medrano-Roldán, Damián Reyes-Jáquez

**Affiliations:** 1 Department of Chemical and Biochemical Engineering, National Technological Institute of Mexico (TecNM)—Durango Institute of Technology (ITD), Blvd. Felipe Pescador 1830, Nueva Vizcaya, Durango 34080, Durango, Mexico; 17040791@itdurango.edu.mx (D.L.A.-B.); 11041103@itdurango.edu.mx (J.I.G.-B.); hiramdurango@yahoo.com.mx (H.M.-R.); 2 Department of Biology, Eastern New Mexico University, 1500 S Ave K, Portales, NM 88130, USA; cristian.cabralesarellano@enmu.edu; 3 Food Science and Technology, Department of Family and Consumer Sciences, New Mexico State University, P.O. Box 30001, Las Cruces, NM 88003, USA; edelgad@nmsu.edu; 4 SECIHTI-Instituto de Silvicultura e Industria de la Madera, Universidad Juárez del Estado de Durango, Blvd. Guadiana No. 501, Cd. Universitaria, Durango 34120, Durango, Mexico; gerardo.pamanes@gmail.com

**Keywords:** RNA aptamer, molecular docking, ACC synthase, ACC oxidase

## Abstract

Worldwide, 13.3% of food was wasted in 2020. Ethylene biosynthesis, responsible for fruit ripening, regulates key processes in plant growth and aging. Aptamers are DNA or RNA molecules with the capacity to bind with high affinity and specificity to proteins due to their three-dimensional structure. Therefore, conventional aptamer selection methods are often costly, inefficient, and time-consuming. In this context, in silico molecular docking offers an efficient alternative, enabling the evaluation of binding potential prior to experimental assays. This research identified aptamers with high predicted affinity for the 1-aminocyclopropane-1-carboxylate synthase (ACC synthase) and 1-aminocyclopropane-1-carboxylate oxidase (ACC oxidase) enzymes, essential in ethylene biosynthesis. Using ZDOCK for preliminary screening and HDOCK for refined analysis, aptamer-enzyme interactions were modeled. Aptamers AB451 and ABR6P.1 showed promising binding to ACC synthase, while RO33828 and O0O6O1 were optimal for ACC oxidase. These results represent a computational foundation for the development of aptamer-based inhibitors to potentially delay ripening and reduce postharvest losses. Experimental validation will be required to confirm their inhibitory function.

## 1. Introduction

The ripening stage of fruits is key for their storage, transportation, and shelf life, as well as for the quality perceived by the consumer. This quality is defined by sensory, nutritional, chemical, and mechanical characteristics, as well as possible defects that affect its consumption or conservation [1]. To evaluate ripeness, physicochemical changes during development and postharvest are analyzed. Among the main indicators are size, shape, flesh firmness, soluble solids content, acidity, and color, as these factors influence the texture, flavor, and appearance of the fruit [2]. Ethylene is a gaseous phytohormone that influences growth, development, and stress response in plants. It plays a key role in fruit ripening, flower aging, and the regulation of processes such as germination and senescence [3,4]. Due to its simple chemical structure and its ability to act at low concentrations, it functions without the need of a transport system, since it can exert its effect in the same place where it is produced [5].

Ethylene biosynthesis begins with the conversion of methionine to S-adenosylmethionine (SAM). Next, ACC synthase transforms SAM into 1-aminocyclopropane-1-carboxylate (ACC) and methylthioadenosine (MTA), the recycling that maintains methionine levels. Finally, ACC oxidase converts ACC to ethylene, carbon dioxide, and cyanide, which is metabolized by β-cyanoalanine synthase. In addition, part of ACC can be converted to malonyl-ACC, regulating ethylene production [3,6]. Although ACC formation is usually considered the limiting step in this pathway, there are situations in which ACO is absent, and both ACS and ACO are induced, as occurs in response to wounding or during stimulated ripening [3]. Given their central role in ethylene biosynthesis, ACC synthase and ACC oxidase represent potential molecular targets for controlling fruit ripening. Ethylene regulates plant development, senescence, and ripening, thus controlling its biosynthesis can directly affect postharvest quality.

Aptamers are short sequences of DNA or single-stranded RNA that can bind with high affinity and specificity to proteins or other molecules. Their three-dimensional structure allows them to recognize specific targets and induce effects such as inhibition or activation. They are characterized by their stability, rapid production, low cost, and ability to recover after denaturation. In addition, their structural flexibility and resistance to temperature changes make them ideal for various scientific and medical applications [7,8,9].

Aptamers are designed to recognize specific target molecules through distinct three-dimensional structures formed by their nucleotide sequences. This specificity is a critical feature that distinguishes aptamers from other classes of ligands, such as antibodies. Research has shown that aptamers can achieve high levels of affinity (from picomolar to nanomolar ranges) towards specific targets, such as proteins and small molecules, due to non-covalent interactions including hydrogen bonds and van der Waals forces [10,11]. These interactions are highly dependent on the exact sequence and length of the aptamers, as even slight variations can significantly impact their binding characteristics [12].

However, the selection of effective aptamers is hindered by the structural diversity inherent to these molecules. In silico docking provides a preliminary platform to predict aptamer-target affinity, reducing time and cost before committing to experimental selection methods.

In silico molecular docking uses computational simulations to analyze the interaction between nucleic acids and their targets. In aptamer design, this technique allows the identification of key structures that enhance their binding to specific molecules, optimizing their affinity and efficiency [13,14]. Different software have been used in the design, structure prediction, molecular docking, and aptamer selection such as: GRAMM, ZDOCK, HDOCK, RNAcomposer, HADDOCK, Mfold, among others [15,16,17,18,19].

The objective of this research was to evaluate the in silico affinity of RNA aptamers for ACC synthase and ACC oxidase, two key enzymes involved in ethylene biosynthesis. Since ethylene plays a central role in fruit ripening, targeting its biosynthetic pathway with aptamers could serve as a potential inhibitory strategy, contributing to broader efforts in ethylene regulation.

## 2. Results

### 2.1. 1-Aminocyclopropane-1-Carboxylate Synthase

ACC synthase is the enzyme that catalyzes the conversion of S-adenosyl methionine to 1-aminocyclopropane-1-carboxylic acid, which is the precursor of ethylene. Four models of the following species were analyzed: tomato (P18485 and Q42881), apple (P37821), and pumpkin (Q00257). 

#### 2.1.1. Amino Acid Composition

Information from four models extracted and modeled from different organisms according to the UniProt database [20] was analyzed as shown in Table 1.

It should be noted that despite being different models, most of the amino acids mostly coincide in leucine, serine, and lysine. The complete amino acid composition is shown in Table 2.

#### 2.1.2. Binding Sites

The relevant characteristics reported for the ACC synthase enzyme include the substrate binding sites. Table 3 shows the amino acids and their positions.

**Figure 1:**
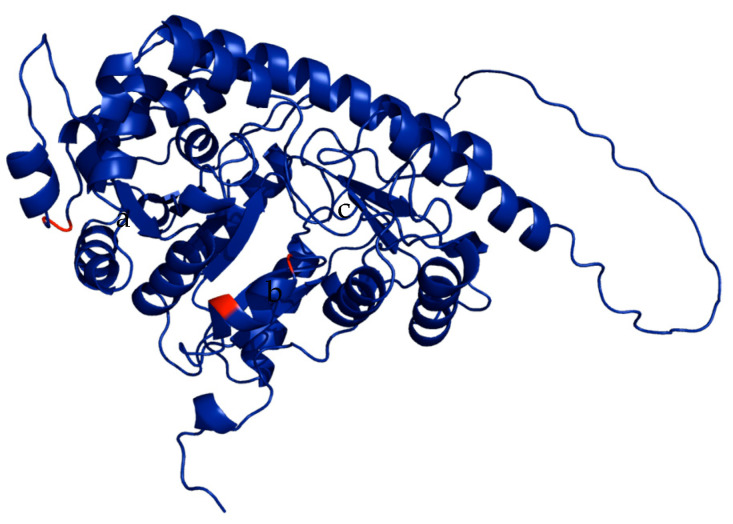
ACC synthase enzyme model P37821, (a) Asp-84 and Tyr-85, (b) Tyr-145, (c) Asp-151. Visualization in PyMol program.

Based on the confidence of the enzyme model of the UniProt database, the model with primary input P37821 was used, which can be seen in Figure 1.

### 2.2. 1-Aminocyclopropane-1-Carboxylate Oxidase

The enzyme 1-aminocyclopropane-1-carboxylate oxidase (ACC oxidase) is the enzyme that catalyzes the reaction that converts 1-aminocyclopropane-1-carboxylic acid to ethylene. The four species analyzed were avocado (P19464), apple (O48882), tomato (P24157), and banana (Q9FR99).

#### 2.2.1. Amino Acid Composition

Information from 4 models extracted and modeled from different organisms according to the UniProt database [20] was analyzed as shown in Table 4.

Among the different models, the majority of amino acids coincide mostly being leucine, glutamic acid, and lysine. The complete amino acid composition is shown in Table 5.

#### 2.2.2. Bonding Sites

Within the typical characteristics of the ACC oxidase enzyme, it is important to identify relevant binding sites as cofactor binding sites as shown in Table 6.

**Figure 2:**
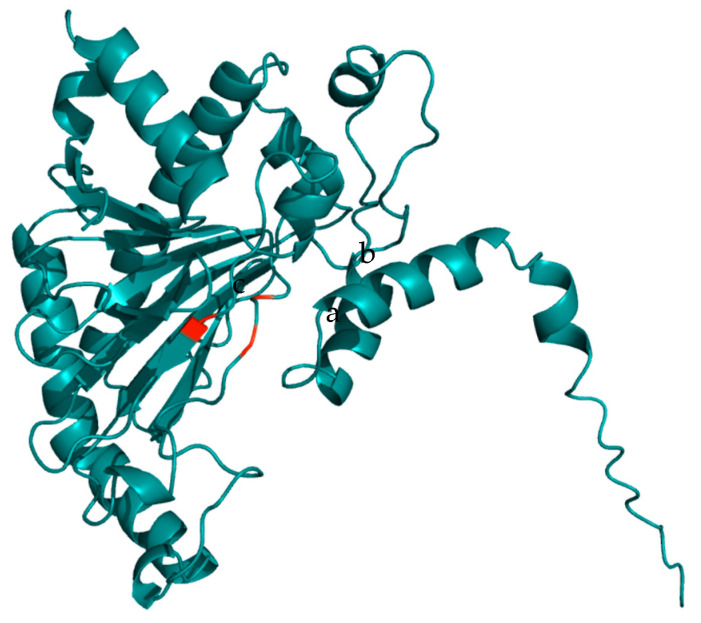
ACC oxidase enzyme model P37821, (a) His-177, (b) Asp-179, (c) His-234. Visualization in PyMol program.

It can be observed that, in the different models, the amino acids involved in binding to the cofactor coincide, and the position of the amino acid varies only in one model. According to the reliability of the three-dimensional model of the enzyme reported in the database, the model with the primary input O48882 was selected, as seen in Figure 2.

### 2.3. Preselection of Aptamers

Based on the characteristics of the target enzymes, aptamers already reported for specific amino acids or for proteins rich in certain amino acids were taken as a starting point for the selection of aptamers. These were consulted in the AptaBase database, which can be consulted in Table 7.

**Figure 3:**
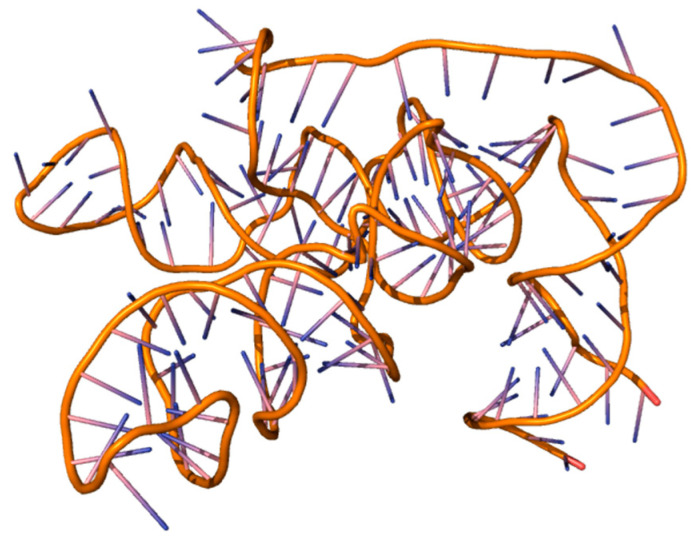
Aptamer AB451, visualization in PyMol program.

For example, the aptamer AB328 (5′ GGCAUCGGAAAGUGGGUUGAUGUAAGUAAGUAACAGGCGAUGCC 3′) has a high affinity for L-histidine (Figure 3). The aptamer sequences were modeled through the RNA composer server with the RNAstructure tool.

### 2.4. In Silico Molecular Docking

#### 2.4.1. ZDOCK Server: First Selection

A total of 116 aptamers were used (including the seven extracted from AptaBase). New aptamers were generated based on the preselected ones obtained from the AptaBase database. The ZDOCK server was used as a first step for option reduction due to its docking algorithm.

The aptamers were evaluated on both enzymes, and once these results were obtained, the most favorable values (highest ZDOCK score) for aptamer-enzyme binding were selected for a second round of molecular dynamics analysis. Table 8 shows the aptamers selected for the second analysis using HDOCK server.

**Figure 4:**
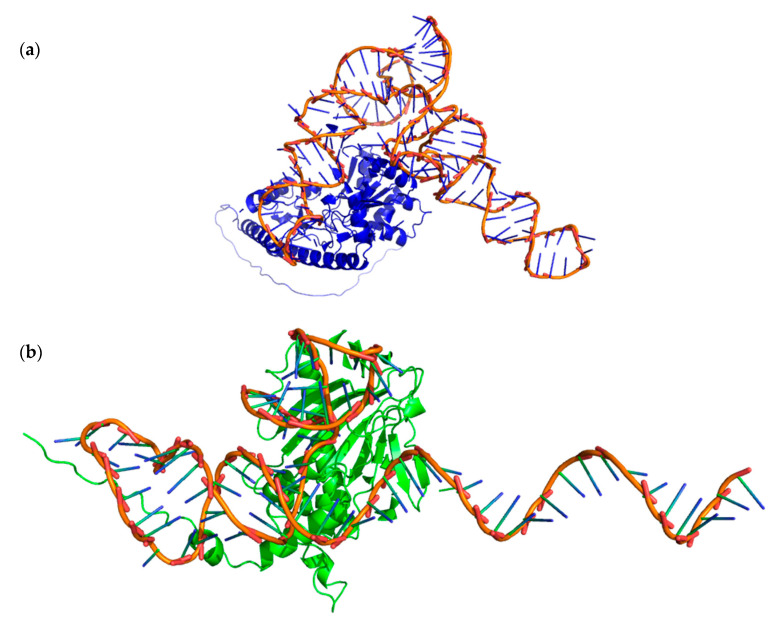
Enzyme–Aptamer interactions obtained at ZDOCK. (**a**) ACC synthase-aptamer AB451, (**b**) ACC oxidase-aptamer AB382 visualization in PyMol program.

In Figure 4, you can see the aptamer-enzyme interaction obtained in ZDOCK server.

In 2021, they used RNA aptamers against the M^pro^ protein of SARS-CoV-2, where the ten best interactions obtained in the ZDOCK server reached values from 1615.67 to 1738.34 [8]. In another study where a carcinoembryonic antigen was targeted, different DNA aptamer structures were analyzed where ZDOCK score values of up to 333.443 were obtained [22]. In a different study, where DNA aptamers were also used, in this case against a specific prostatic antigen, the selected optimal aptamer obtained a value of 42.64 in ZDOCK score [23].

#### 2.4.2. HDOCK Server: Second Selection

A second evaluation was performed to determine the optimal aptamers for the target enzymes. A total of 20 aptamers that scored the highest on the ZDOCK server were evaluated. The docking score and confidence values are shown in Table 9 and Table 10.

**Figure 5:**
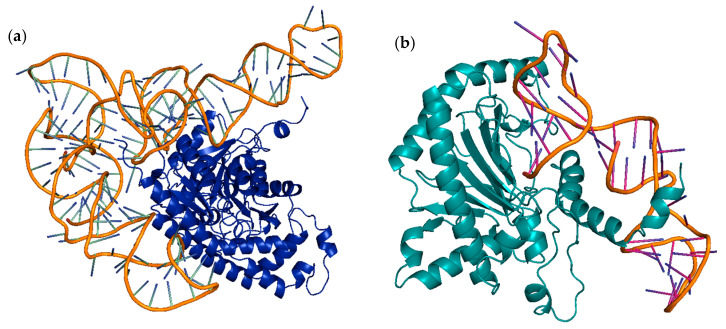
Enzyme–Aptamer interactions obtained at HDOCK. (**a**) ACC synthase-aptamer AB451, (**b**) ACC oxidase-aptamer AB382 visualization in PyMol program.

In Figure 5, you can see the aptamer-enzyme interaction obtained in HDOCK server.

It can be seen in Table 5, Table 6 and Table 7 that the relative positions of the aptamers in the lists vary between the evaluations performed by the different servers. For example, with the ACC synthase enzyme, the ABR7 aptamer is found in position 14 on the HDOCK server but on the ZDOCK server it is found in position 2. These differences reflect how each tool weighs the affinity and structural complementarity criteria. This comparative approach ensures a robust analysis to determine the best candidates.

## 3. Discussion

This work presents a computational strategy for identifying RNA aptamers with potential affinity for key enzymes in ethylene biosynthesis.

The inclusion of amino acid composition data for different modeled forms of ACC synthase and ACC oxidase enzymes serves multiple complementary purposes within the context of this study. First, amino acid composition is a foundational descriptor of protein properties, influencing structural stability, folding behavior, catalytic activity, and interaction specificity [24]. By quantifying and comparing composition across enzyme models, we provide additional insight into the biochemical variability that may arise from isoform-specific sequences, tissue-specific expression, or species-specific divergence [25].

Second, the comparative analysis of amino acid content across enzyme models enables an indirect evaluation of model plausibility and functional conservation. For instance, conserved profiles in hydrophobicity, charged residues, or specific functional residues (e.g., cysteines, histidines, or aromatic residues) can support the biological validity of the predicted models, particularly when experimental structural data are unavailable [26]. Differences may indicate functional specialization or varying post-translational modification potentials among enzyme variants [27]. Studies have demonstrated that the presence of certain amino acids can significantly impact protein functionality and interaction dynamics, emphasizing the importance of examining amino acid composition in evolutionary and bioengineering contexts [28].

Moreover, amino acid composition metrics are useful for guiding downstream computational or experimental strategies. For example, differences in amino acid usage can inform docking strategies, particularly when evaluating surface residues involved in protein-ligand or protein–RNA interactions. They also contribute to predicting solubility, stability, and aggregation propensity—important considerations for future in vitro expression or mutagenesis studies [29,30]. Our previous work supports the notion that specific amino acid profiles can influence the thermal stability and aggregation behavior of proteins, providing a rationale for this analysis [31].

The three-dimensional model selected for the ACC synthase enzyme shows relevant binding sites on aspartate (Asp) and tyrosine (Tyr) residues, while the predominant amino acids are leucine (Leu), serine (Ser), and glutamate (Glu). Similarly, the model for the ACC oxidase enzyme evidence key binding sites in histidine (His) and aspartate (Asp), with the predominance of leucine (Leu), lysine (Lys), and glutamate (Glu).

In the context of the enzyme 1-aminocyclopropane-1-carboxylate synthase (ACC synthase), which catalyzes the conversion of S-adenosyl methionine to 1-aminocyclopropane-1-carboxylic acid, aptamers can target specific regions or motifs within this enzyme. ACC synthase from different species, including tomato, apple, and pumpkin, can potentially be recognized by aptamers designed to interact with key amino acid sequences critical for the enzyme’s catalytic function or structural stability. The references regarding these specific enzymes and aptamer interactions are only partially supportive of these claims; for example, studies have demonstrated the biochemical activity of ACC synthase but not directly the specific interactions with aptamers related to sequence targeting [32,33].

The UniProt database serves as a valuable resource for examining protein sequences and their related functional annotations to identify potential binding sites for aptamers. Through tools such as BLAST 2.16.0, researchers can analyze the sequences of ACC synthase and determine conserved motifs that may be amenable to aptamer binding, thus enhancing the specificity and efficacy of these nucleic acid-based binders in biochemical applications. This is supported by the fact that UniProt provides detailed annotations regarding protein domains and sequences, although the specific methodology of using tools like BLAST for aptamer binding needs to be discussed more precisely [34,35]. Furthermore, understanding the interaction between aptamers and specific amino acid sequences in proteins like ACC synthase is pivotal for advancing diagnostics and therapeutic strategies that leverage these unique interactions.

For in silico molecular docking analysis to determine which aptamer is more optimal, two online servers were used: ZDOCK server (3.0.2. version) and HDOCK server. Both ZDOCK and HDOCK utilize advanced numerical methods to enhance their performance. ZDOCK, for instance, leverages the FFT algorithm to accelerate the search for optimal docking orientations, significantly reducing computational time while maintaining accuracy [36,37]. HDOCK, in contrast, employs a combination of Monte Carlo sampling and molecular dynamics simulations to explore the conformational space more thoroughly, which is particularly beneficial for capturing the dynamics of protein interactions [38,39].

After an initial in silico simulation on the ZDOCK server, it was possible to make a first selection of the 20 most optimal aptamers for a second simulation on the HDOCK server. In Table 5, Table 6 and Table 7, it is observed that the values obtained using the HDOCK server exceed the standards generally reported for protein–RNA complexes, where a typical docking score is around −200. Furthermore, in terms of confidence score, a value higher than 0.7 indicates a high probability of binding. It is noteworthy that the aptamers with the best performance in both enzymes reach a confidence score of 0.99, evidencing their potential as optimal candidates [40].

In the HDOCK server, both the docking score and confidence score are essential for evaluating the quality of predicted molecular interactions. The docking score, which is generated through a knowledge-based scoring function (ITScore-PP), provides a dimensionless metric where more negative values signify stronger binding affinities between the interacting molecules [41]. This score allows researchers to rank docking poses based on their predicted stability and viability. Complementarily, the confidence score reflects the reliability of these docking predictions, indicating the likelihood that the observed interactions are consistent with biochemical realities. A confidence score above 0.7 is generally considered a strong indicator of potential binding, providing an additional layer of validation for the docking models produced [42]. Together, these scores enable researchers to make informed decisions regarding the best models for further investigation, enhancing the predictive power of molecular docking studies.

ZDOCK is recognized for its scoring capability in molecular docking, particularly in analyzing binding modes and affinities, which supports its use as an initial screening tool for identifying potential aptamer–enzyme interactions [43,44,45].

The choice of both ZDOCK and HDOCK is grounded in the comparative ranking ability of their scoring functions, which have been shown to effectively distinguish between high-affinity and low-affinity interactions within a large pool of candidates [44,46]. HDOCK complements ZDOCK by providing additional accuracy through flexibility in receptor and ligand conformations, which is crucial for capturing the dynamics of aptamer-binding interactions [47,48,49]. Validation studies confirm these tools’ capabilities to identify true binding ligands from extensive candidate libraries, reinforcing their application in virtual screening processes [47].

Furthermore, molecular docking serves as a significant component in the rational design of binding aptamers. Accurate predictions of binding affinities are essential for optimizing lead candidates. Various studies illustrate that correctly applied docking tools can enhance the predictive power related to binding affinities, a key requirement in aptamer selection methodologies [48,50,51]. Particularly, the high-performance scoring metrics generated by these docking tools have been highlighted to closely align with experimental binding affinities, which adds confidence in the results produced from the docking screens [52,53].

According to the docking score analysis performed with the ZDOCK server for the first analysis and initial screening, and a second analysis in HDOCK, the AB451 aptamer (5′ AGUAAUACGACUCACUAUAGGGAGAAUUCCGACCAGAAGUUGGCGUUGGCAUGACGCGGGGAAUCGGGUGCAUCGAUGACUACUCCUGGGCCCACGUCUGUUGUUGACGUCACAGCUUGAUUUAGGAUAGCGCUUGGGCAGUCGUGCAGUGGA 3′) showed the highest affinity for ACC synthase, whereas for ACC oxidase the RO33828 aptamer (5′ GACGAGAAGGUACUAGCAGGUAGGUCACUCGUCGGCAUCGCGAUGCC 3′) was the optimal. These results reflect a high binding affinity, suggesting that these aptamers could play a relevant role in the control of enzyme activity. The evaluation on two molecular docking platforms allowed to reduce the number of options and to increase the reliability of the obtained results [8].

Aptamers generally achieve binding specificity through a combination of molecular shape complementarity, electrostatic interactions, stacking interactions, and hydrogen bonds [54]. Each of these factors contributes to the overall binding affinity and specificity of the aptamer for its target protein.

When analyzing the enzyme 1-aminocyclopropane-1-carboxylate synthase (ACC synthase), the docking approach allows the identification of specific regions on the protein that are critical for aptamer binding. For instance, conserved amino acid sequences or catalytic sites within the ACC synthase structure may present favorable interactions for aptamer attachment. As demonstrated in studies involving docking analyses, specific residues can form hydrogen bonds with the aptamer, which play a pivotal role in stabilizing the aptamer-protein complex [55,56].

The HDOCK server, employed in our docking assessments, effectively predicts the binding modes of aptamer-protein interactions by evaluating the conformations that yield optimal fits and energy scores. HDOCK is a hybrid docking approach that combines template-based modeling with free docking information, predicting binding modes and three-dimensional structures of protein complexes by identifying best-fitting conformations [57]. This approach enhances the accuracy of predicting how specific structural motifs of ACC synthase may interact with aptamers, thereby guiding the identification of likely binding sites [58].

Moreover, it is essential to note that the potential influence of amino acid mutations in critical regions, as observed with aptamer–target interactions, can drastically alter the binding dynamics. For example, changes in the residues forming the binding pocket can result in reduced aptamer affinity, indicating the significance of targeting stable regions or critical residues on proteins when designing aptamers for therapeutic applications [59].

In this study, we adopted a rigid-body docking strategy using RNAComposer, ZDOCK, and HDOCK to model protein–RNA interactions. We fully acknowledge the critical importance of RNA conformational flexibility—particularly for aptamers with substantial single-stranded regions—but the chosen methodology reflects a balance between biological realism, computational tractability, and reproducibility. The incorporation of RNAComposer allowed us to generate 3D models constrained by secondary structure predictions and thermodynamic stability, aligning our approaches with experimentally informed folding principles [60]. Although these models provide only a single conformation from RNA’s broader structural ensemble, they serve as biologically plausible initial structures rather than arbitrary models and are widely accepted in the field for exploratory docking studies.

The docking pipeline combines ZDOCK and HDOCK to leverage their respective strengths. ZDOCK is known for its efficient global sampling based on geometric complementarity and electrostatics, making it effective as a first-stage filter for identifying candidate binding modes [61]. HDOCK subsequently refines these results using a hybrid scoring system that employs knowledge-based statistical potentials along with energy terms, which has been shown to enhance the accuracy and reliability of complex models [62,63]. This sequential approach is not only complementary in evaluating interactions but also mitigates scoring bias inherent to any single tool, which is crucial given the nuances of RNA-protein interactions [64].

While both ZDOCK and HDOCK implement rigid docking frameworks, their integration has been successfully applied in previous studies [65]. This provides a reasonable first approximation of interaction geometry, which is essential for generating testable hypotheses rather than delivering definitive high-resolution models of RNA-protein complexes. We fully acknowledge that the lack of RNA conformational sampling may limit the accuracy of specific binding predictions; however, this limitation has been explicitly discussed in the manuscript. Given the computational demands associated with flexible docking methodologies or molecular dynamics-based ensemble approaches, we consider our pipeline to be suitable for the exploratory scope of this study. Furthermore, this strategy lays a reproducible and structured foundation for future refinements using more advanced modeling techniques or experimental validation, ensuring that the insights gained from our models are meaningful and actionable.

Nonetheless, these results represent a preliminary computational assessment and cannot confirm functional inhibition. RNA flexibility was not included in this phase; this limitation can influence the accuracy of docking results. Future studies will address this and enrich the docking prediction. As a future perspective, it is proposed to perform protein–RNA interaction assays to experimentally validate the binding of the aptamers to the target enzymes and confirm the results of the simulations. For aptamers that demonstrate a favorable result in these initial assays, it is suggested to perform additional tests that evaluate their effect on enzyme activity. These tests would consist of incorporating the aptamers during enzyme-catalyzed reactions to determine their inhibitory or modulating capacity.

## 4. Materials and Methods

The in silico selection of aptamers for specific selected targets is summarized in the next steps:

1. Molecular target identification and recognition.

Firstly, it is necessary to identify the target molecules to identify the most relevant structural and typical characteristics. In summary, ethylene is produced from methionine by three fundamental enzymatic reactions: methionine is converted to S-AdoMet by the action of S-AdoMet synthetase; ACC synthase converts S-AdoMet to ACC; and ACC oxidase degrades ACC, releasing ethylene. Although ACC formation is usually considered the limiting step in this pathway, there are situations in which ACC oxidase is absent, and both ACC synthase and ACC oxidase are induced, as occurs in response to wounding or during stimulated ripening [3,6].

Based on this information, ACC synthase and ACC oxidase enzymes were selected as molecular targets for this study. The three-dimensional structure extracted from different organisms was analyzed to identify differences, similarities, amino acid composition and relevant binding sites.

2. Pre-selection of aptamers.

Based on the characteristics of the target enzymes, aptamers are taken as a starting point for the selection of aptamers. Testing started with aptamers already reported for specific amino acids or for proteins rich in certain amino acids.

3. Three-dimensional modeling of aptamers.

Only RNA aptamers were used for this study. To perform the three-dimensional modeling of the aptamers from their sequence, the RNAComposer web server was used with the RNAStructure tool as a secondary structure prediction method.

RNAComposer is a tool that predicts the three-dimensional structure of RNA in an automated or semi-automated way. It accepts as input RNA sequences and, optionally, their secondary structure in bracket notation. If not provided, it automatically predicts it with integrated software. It operates in two modes: interactive, to process a single data set and generate a 3D model, and batch, which allows multiple sequences to be analyzed and custom configurations to be made [66].

RNAComposer is a bioinformatics tool that combines a computational core, a database of 3D RNA structures and a web interface. It integrates external software to predict secondary structures, model 3D elements, and optimize conformation by energy minimization. This architecture provides accurate models of RNA structure [67].

4. In silico molecular docking.

Molecular docking is a process that combines sampling and evaluation. Starting from two individual structures, this method explores all possible modes of interaction between the two structures. Subsequently, a scoring function is applied to rank the identified binding modes, either during the sampling process or upon completion [62].

ZDOCK is a molecular docking program that uses a Fast Fourier Transform (FFT) algorithm to search for interactions in three-dimensional space. Its scoring system considers shape complementarity, electrostatic interactions, and statistical potential. The prediction process includes three stages: structure input, key residue selection, and result visualization [68]. The ZDOCK program uses a gridded representation of the two proteins together with a three-dimensional Fast Fourier Transform (FFT) to efficiently explore the search space for rigid-body docking positions [69].

ZDOCK’s Fast Fourier Transform (FFT)-based sampling enables exhaustive global conformational searches, facilitating high-throughput generation of plausible poses based on shape complementarity and electrostatics. This attribute renders ZDOCK particularly effective as a pre-filter to narrow down candidate poses from a vast conformational space with minimal computational cost, as supported by studies highlighting the efficacy of FFT approaches in enhancing docking performance [70].

ZDOCK operates primarily on the principles of rigid-body docking, utilizing a Fast Fourier Transform (FFT) algorithm to efficiently search the conformational space of protein complexes. The core of ZDOCK’s methodology involves discretizing the protein structures into three-dimensional (3D) voxel representations, which allows for rapid calculations of shape complementarity and electrostatic interactions between the proteins [36,37,71]. The docking process begins with the generation of many potential orientations and positions for the protein components, which are then evaluated using a scoring function that incorporates pairwise shape complementarity, electrostatics, and interface atomic contact energy [36,37,71]. The scoring function is crucial as it quantifies how well the two proteins fit together, with higher scores indicating better predicted interactions.

The ZDOCK algorithm is based on the Fast Fourier Transform (FFT) and is summarized in the following steps: the central receptor is cordoned at the origin according to the center of mass; the central ligand is cordoned at the origin according to the center of mass; the cubic grid size is selected to contain centered molecules for FFT and finally a 3D FFT is performed to calculate the convolution between the ligand and receptor grids, and the highest scoring position of the resulting grid is selected; between these steps, discretization protocols occur that incorporate the ZDOCK scoring function [69].

The mathematical foundation of ZDOCK’s scoring function can be expressed in terms of several components. The shape complementarity score (PSC) evaluates the geometric fit of the two proteins, while the electrostatic score accounts for the interactions between charged residues [36,71]. The interface atomic contact energy (IFACE) score further refines the predictions by assessing the energy associated with the atomic contacts at the interface of the two proteins [37,71]. The overall ZDOCK score is a combination of these individual scores, which allows for a comprehensive evaluation of the docking poses generated during the search process.

In the output data, the predictions are ordered according to the ZDOCK score, the predictions considered to be the best are at the top being the highest ZDOCK score values, which vary by molecule and ligand [68].

HDOCK is a server for molecular docking between proteins and RNA/DNA, using 3D structures when available. It can assign these molecules as receptor or ligand and automatically identifies their types to apply the appropriate method. For protein-RNA/DNA, it uses FASTA100 to search for homologous templates and a specific scoring function. For protein–protein, it combines template-based modeling with *ab initio* docking [37].

HDOCK, on the other hand, incorporates a hybrid scoring function that blends knowledge-based statistical potentials, empirical energy terms, and template-based constraints. The strength of HDOCK lies in its ability to re-rank and refine the pre-filtered models by evaluating the interface quality more comprehensively. Knowledge-based scoring functions are recognized for their reduced steepness compared to empirical scoring functions, which leads to a more robust performance against conformational variations [72]. By leveraging ZDOCK’s broad sampling capabilities alongside HDOCK’s refined scoring mechanisms, we aim to enhance both efficiency and predictive accuracy in our docking process [73].

HDOCK optimizes the scoring of protein-RNA interactions using ITScore-PR, a method based on statistical mechanics and known complex structures. It uses a global docking approach with Fast Fourier Transform (FFT) to explore possible binding modes. Its workflow includes four stages: data input, sequence similarity search, structural modeling, and global docking, prioritizing user-supplied structures [74].

HDOCK, conversely, builds upon the rigid-body docking approach but incorporates additional flexibility and scoring refinements. HDOCK integrates both template-based modeling and energy-based scoring to enhance the accuracy of its predictions. It employs a hybrid strategy that combines rigid-body docking with flexible refinement, which is particularly useful for accommodating the inherent flexibility of protein structures during interactions [38,39]. HDOCK’s methodology involves an initial rigid-body docking phase, followed by a flexible refinement stage that optimizes the docked conformations based on energy minimization techniques [14,19].

In the evaluation metrics, the docking score is calculated by an iterative scoring function that is based on knowledge of the ITScorePR; a more negative docking score means a more likely binding model; however, since protein–RNA complexes usually have a docking score of around −200, HDOCK has empirically defined a confidence score as shown in Equation (1) to indicate the probability of binding of the two interacting molecules, in which a value less than 0.5 is unlikely to bind, a value between 0.5 and 0.7 is likely to bind, and a value greater than 0.7 is very likely to bind [75].

(1) Confidence score=1.0[1.0+e0.02∗Docking Score+150]

The scoring mechanism in HDOCK is also multifaceted, incorporating energy terms that account for van der Waals interactions, electrostatics, and solvation effects. This comprehensive scoring approach allows HDOCK to evaluate the stability of the predicted complexes more effectively than traditional rigid-body docking methods [21,22]. The docking score in HDOCK is indicative of the predicted binding affinity, with lower energy scores suggesting more favorable interactions between the protein partners.

Following the initial docking analysis with ZDOCK, the top-ranked aptamer–enzyme complex was selected and submitted to the HDOCK server for a second round of docking. No residue-based restraints were applied during this step; the docking was carried out in fully blind mode to allow the algorithm to explore potential binding interactions without predefined biases. The HDOCK server provided a docking score and a confidence score for each predicted pose. These values were used to assess the plausibility of the redocked complex. RMSD values with respect to the ZDOCK-generated input pose were not available from the HDOCK server and thus were not included in the analysis.

## 5. Conclusions

The three-dimensional model of ACC synthase enzyme shows key binding sites on aspartate (Asp) and tyrosine (Tyr) residues, with predominance of leucine (Leu), serine (Ser) and glutamate (Glu). Similarly, ACC oxidase presents relevant sites in histidine (His) and aspartate (Asp), with leucine (Leu), lysine (Lys), and glutamate (Glu) as predominant amino acids.

Molecular docking analysis performed in ZDOCK and validated with HDOCK identified aptamer AB451 as having the highest affinity for ACC synthase, while RO33828 was the most suitable for ACC oxidase. The combination of these two tools allowed filter options and improve the accuracy of the results, suggesting that these aptamers could influence the regulation of enzyme activity.

While the study is purely in silico, its aim was to pre-select aptamer candidates using robust computational criteria, enabling a rational reduction in possibilities before advancing to experimental validation.

## Figures and Tables

**Table 1:** Amino acids of different enzyme models of the enzyme ACC synthase.

Amino Acids/Model	P18485 *	P37821 *	Q00257 *	Q42881 *
Leucine	42	49	46	49
Serine	41	42	47	45
Lysine	35	26	33	33
Alanine	28	31	32	29
Glutamic acid	28	32	30	29
Glycine	30	31	28	28

* Primary access to UniProt database.

**Table 2:** Amino acid composition.

	1-Aminocyclopropane-1-Carboxylate Synthase				Average	Percentage
Amino Acids/Model	P18485	P37821	Q00257	Q42881		
A (Alanine)	28	31	32	29	30	6.31
C (Cysteine)	11	9	9	9	9.5	2.00
D (Aspartic acid)	29	20	21	23	23.25	4.89
E (Glutamic acid)	28	32	30	29	29.75	6.26
F (Phenylalanine)	29	24	27	26	26.5	5.57
G (Glycine)	30	31	28	28	29.25	6.15
H (Histidine)	9	15	11	9	11	2.31
I (Isoleucine)	29	19	22	21	22.75	4.78
K (Lysine)	35	26	33	33	31.75	6.68
L (Leucine)	42	49	46	49	46.5	9.78
M (Methionine)	14	13	14	15	14	2.94
N (Asparagine)	30	28	29	34	30.25	6.36
P (Proline)	24	19	14	15	18	3.79
Q (Glutamine)	13	16	17	16	15.5	3.26
R (Arginine)	24	23	22	22	22.75	4.78
S (Serine)	41	42	47	45	43.75	9.20
T (Threonine)	20	23	26	21	22.5	4.73
V (Valine)	30	30	27	23	27.5	5.78
W (Tryptophane)	7	8	8	8	7.75	1.63
Y (Tyrosine)	12	15	12	14	13.25	2.79
TOTAL	485	473	475	469	475.5	100

Primary access to UniProt database.

**Table 3:** ACC synthase enzyme binding sites.

P18485	P37821
Glutamic acid 55, substrate	Aspartic acid 84, substrate
Tyrosine 92, substrate	Tyrosine 85, substrate
	Tyrosine 145, substrate
	Aspartic acid 151, substrate

Primary access to UniProt database.

**Table 4:** Amino acids of different enzyme models of the enzyme ACC oxidase.

Amino Acids/Model	O48882 *	P19464 *	P24157 *	Q9FR99 *
Leucine	33	32	33	25
Glutamic acid	30	33	29	28
Lysine	29	26	26	22
Alanine	22	23	20	25
Valine	17	26	21	21
Aspartic acid	21	18	21	22

* Primary access to UniProt database.

**Table 5:** Amino acid composition.

	1-Aminocyclopropane-1-Carboxylate Oxidase				Average	Percentage
Amino Acids/Model	O48882	P19464	P24157	Q9FR99		
A (Alanine)	22	23	20	25	22.5	7.08
C (Cysteine)	3	4	4	4	3.75	1.18
D (Aspartic acid)	21	18	21	22	20.5	6.45
E (Glutamic acid)	30	33	29	28	30	9.43
F (Phenylalanine)	16	17	15	15	15.75	4.95
G (Glycine)	20	18	19	23	20	6.29
H (Histidine)	8	7	7	6	7	2.20
I (Isoleucine)	17	11	15	14	14.25	4.48
K (Lysine)	29	26	26	22	25.75	8.10
L (Leucine)	33	32	33	25	30.75	9.67
M (Methionine)	11	15	12	8	11.5	3.62
N (Asparagine)	15	15	16	10	14	4.40
P (Proline)	17	17	17	19	17.5	5.50
Q (Glutamine)	8	8	11	11	9.5	2.99
R (Arginine)	13	11	11	17	13	4.09
S (Serine)	18	13	14	13	14.5	4.56
T (Threonine)	17	15	11	9	13	4.09
V (Valine)	17	26	21	21	21.25	6.68
W (Tryptophane)	4	3	3	5	3.75	1.18
Y (Tyrosine)	11	8	11	9	9.75	3.07
TOTAL	330	320	316	306	318	100

Primary access to UniProt database.

**Table 6:** ACC oxidase enzyme binding sites.

O48882 *	P19464 *	P24157 *	Q9FR99 *
Histidine 177, Cation Fe (Cofactor)	Histidine 178, Cation Fe (Cofactor)	Histidine 177, Cation Fe (Cofactor)	Histidine 177, Cation Fe (Cofactor)
Aspartic acid 179, Cation Fe (Cofactor)	Aspartic acid 177, Cation Fe (Cofactor)	Aspartic acid 179, Cation Fe (Cofactor)	Aspartic acid 179, Cation Fe (Cofactor)
Histidine 234, Cation Fe (Cofactor)	Histidine 235, Cation Fe (Cofactor)	Histidine 234, Cation Fe (Cofactor)	Histidine 234, Cation Fe (Cofactor)

* Primary access to UniProt database.

**Table 7:** List of pre-selected aptamers.

Aptamer	Reported Affinity	Aptamer Sequence (5′-3′)
AB316	L-Isoleucine	GGUCUUACGUCGUUCGCGACUAUUGGGAGACC
AB328	L-Histidine	GGCAUCGGAAAGUGGGUUGAUGUAAGUAACAGGCGAUGCC
AB338	L-Arginine (44Arg11)	GACGAGAAGGAGCGCUGGUUCUACUAGCAGGUAGGUCACUCGUC
AB382	Phenylalanine	AUUGGAUCGGUAGUAUUUAGGGUGAGACACUUCAUGCCUUUGUUGCAGGCUGGGGUGAAGGCGCUACAUGGCGUCUGAAA
AB391	L-Valine	GGGAGCUCAGAAUAAACGCUCAAAUCCGUGGACAGGGCGUAAGCGCCUUCGACAUGAGACACGGAUCCUGCGACGAAUUCAGC
AB421	L-Arginine (ag.06)	GGAGCUCAGCCUUCACUGCAUGAUAAACCGAUGCUGGGCGAUUCUCCUGAAGUAGGGGAAGAGUUGUCAUGUAUGGGGGCACCACGGUCGGAUCCUG
AB451	D-Tryptophan (MF10)	AGUAAUACGACUCACUAUAGGGAGAAUUCCGACCAGAAGUUGGCGUUGGCAUGACGCGGGGAAUCGGGUGCAUCGAUGACUACUCCUGGGCCCACGUCUGUUGUUGACGUCACAGCUUGAUUUAGGAUAGCGCUUGGGCAGUCGUGCAGUGGA

Note: Data extracted from the Aptabase database. The numbers refer to the position in which they are listed in this database [21].

**Table 8:** Highest values in ZDOCK server.

ACC Synthase		ACC Oxidase	
APTAMER	ZDOCK SCORE	APTAMER	ZDOCK SCORE
AB451	2177.207	AB382	1735.208
ABR7	2074.474	ABR7	1723.118
AB33828	2044.513	AB421P.1	1715.431
O0O6O1	2043.085	RO33828	1679.875
O7O3O5P.2	2032.589	AB451	1672.467
O1P0O0P.1	2029.322	O0O6O1	1659.338
O2O6O4	2024.298	O1O6O0	1618.045
O4O6O2	1948.203	AB33828	1586.102
AB382	1928.178	AB421	1566.979
ABR6P.1	1898.414	APTAR721	1530.529
O1O3O0P.1	1882.831	O5O3O7P.1	1517.614
O2O6O4P.1	1867.794	O4P0O2	1516.753
AB31628	1856.707	O2O6O4	1510.156
O1O3O0	1848.095	AB31628	1503.637
APTAR721P.2	1823.035	O1P0O0P.2	1492.429
O1P0O0	1822.825	AB338	1491.007
O0O6O1P.1	1818.254	O0O3O1	1478.692
AB451P.2	1817.279	O7O3O5	1477.19
O5O3O7	1812.821	ABR482P.1	1472.001
ABR6	1801.812	O5P0O7	1471.887

**Table 9:** ACC enzyme synthase: molecular interaction results obtained from the HDOCK server.

APTAMER	DOCKING SCORE	CONFIDENCE SCORE
AB451	−405.72	0.994
ABR6P.1	−391.45	0.9921
ABR6	−383.2	0.9907
AB33828	−373.59	0.9887
AB31628	−372.65	0.9885
O1O3O0P.1	−368.55	0.9551
AB382	−361.97	0.9858
O5O3O7	−359.29	0.985
O2O6O4	−357.07	0.9843
O1O3O0	−351.71	0.9826

**Table 10:** ACC enzyme oxidase: molecular interaction results obtained from the HDOCK server.

APTAMER	DOCKING SCORE	CONFIDENCE SCORE
RO33828	−382.62	0.9906
O0O6O1	−355.98	0.9763
AB338	−328.09	0.9724
AB451	−327.08	0.9718
AB33828	−317.06	0.9658
ABR7	−306.51	0.9581
AB421P.1	−305.55	0.9573
AB31628	−302.99	0.9552
AB382	−300.27	0.9528
O4P0O2	−299.34	0.952

## Data Availability

The data presented in this study are available on request from the corresponding author.
